# Supplementary material for: Absorbed dose calculation for a realistic CT-derived mouse phantom irradiated with a standard Cs-137 cell irradiator using a Monte Carlo method
Source: PLoS One. 2023 Feb 2;18(2):e0280765. doi: 10.1371/journal.pone.0280765 (PMC9928120; doi:10.1371/journal.pone.0280765)
Supplement: Attachment — Submitted filename: PLOS ONE.docx [file pone.0280765.s002.docx]

The paper entitled " Absorbed Dose Calculation for a Realistic CT-derived Mouse Phantom Irradiated with a Standard Cs-137 Cell Irradiator Using a Monte Carlo Method" has been reviewed for a potential publication in Journal PLOS ONE. The authors develop a CT-derived MC phantom generated from a mouse with a xenograft tumour that used to calculate both the dose heterogeneity in the tumour volume and out of field scattered dose for pre-clinical small animal irradiation experiments. This theoretical method is providing extensive data for the scientific community. Overall the paper is interesting. Most importantly, to ensure that the tumor is treated with the desired dose and that the dose to normal tissue (out of a field) has minimal impact. The titles along with their subtitles are prepared in a proper way. I believe that the provided outcomes would enhance the existing data for preclinical radiobiological research.

However, I have the following recommendations for the authors, which will improve the overall quality of the paper.

*      16, 17 , 19, 21 , 22. 23, 24, 25, 26, 29, 30, 31, 38, 56, 57,58, 62 and 63 references need to be updated. They are all 2010 and older
