# Supplementary material for: Absorbed dose calculation for a realistic CT-derived mouse phantom irradiated with a standard Cs-137 cell irradiator using a Monte Carlo method
Source: PLoS One. 2023 Feb 2;18(2):e0280765. doi: 10.1371/journal.pone.0280765 (PMC9928120; doi:10.1371/journal.pone.0280765)
Supplement: Attachment — Submitted filename: Response to Reviewers.docx [file pone.0280765.s003.docx]

We appreciate the editor and reviewers for taking the time to read and comment on our manuscript. The comments from the reviews are in black and our responses to the reviewers 1, 2, 3, 4 are highlighted in red, blue, green, and brown, respectively. Also, the changes that we have made to the manuscript are all in ***bold italic text*** in both response letter and revised manuscript. In addition to addressing the comments, our recently published study (in Medical Physics) was added to the reference list (see below) and has been cited in lines 102 and 135.

***46. Entezam A, Fielding A, Fontanarosa D. Characterization of a Novel Irradiation Method for Small Animal Radiotherapy Using a Monte Carlo Method. InMEDICAL PHYSICS 2022 Jun 1 (Vol. 49, No. 6, pp. E976-E976). 111 RIVER ST, HOBOKEN 07030-5774, NJ USA: WILEY.***

Reviewer #1: Manuscript cogently describes a study based on the generation of a Monte Carlo model from a micro-CT image set of a mouse with a xenograft tumour for absorbed dose investigations including tumour and out of field doses in the mouse.

1. Line 120-122 - justify the use of these size selections. Are these appropriate for mouse models?

Thank you for the valuable comments. Yes, the selected voxel size is the most appropriate for the mouse model in our study. The following statement that explains the reason for the size selection was added to the manuscript (line 123-126). Also, reference number 51 was added to the manuscript and reference numbers were updated throughout the manuscript accordingly.

***“There is a trade-off between voxel size and statistical accuracy for MC calculations in small animal [32]. The selected voxel size in this study allows for sufficient statistical accuracy while providing adequate resolution for small animal phantoms [32], [51].”***

2. Line 249 - 'countering' should perhaps be 'contouring'?

The word was corrected in line 262 as suggested.

3. Line 261 - refers to 'our radio-immunotherapy investigations', please provide citations to this work.

The first paper for our radio-immunotherapy study is currently still under preparation. However, our previous physics study provides adequate information on the requested citation. So, we provided citations to our previous physics work (reference number 44) in line 274.

4. Table 1 - was precision actually limited to only 1 decimal point for these mean doses? 100 mGy steps seem rather large. Perhaps report to 2 decimals (if measurement method supports). Also mean and range should perhaps be reported here?

Thank you for the comment. We agree with the reviewer that mean doses should be reported to 2 decimals. So, in the revised version, the mean doses in Table 2 are reported with 2 decimals (see below). Also, to include the range, the minimum and maximum dose results are reported in table 2 to present the lower and upper range limits of the doses.

| ***OARs*** | ***Minimum dose (Gy)***  ***Tumour diameter (cm)***  ***0.5 0.75 1*** | | | | ***Maximum dose (Gy)***  ***Tumour diameter (cm)***  ***0.5 0.75 1*** | | | ***Mean dose (Gy)***  ***Tumour diameter (cm)***  ***0.5 0.75 1*** | | | |
| --- | --- | --- | --- | --- | --- | --- | --- | --- | --- | --- | --- |
| ***Rlung*** | ***0.73*** | ***0.72*** |  | ***0.75*** | ***1.35*** | ***1.38*** | ***1.36*** | ***1.10*** |  | ***1.20*** | ***1.21*** |
| ***Llung*** | ***0.84*** | ***0.85*** |  | ***0.79*** | ***1.36*** | ***1.37*** | ***1.33*** | ***1.20*** |  | ***1.21*** | ***1.13*** |
| ***Spine*** | ***0.92*** | ***0.95*** |  | ***0.94*** | ***1.37*** | ***1.36*** | ***1.29*** | ***1.14*** |  | ***1.24*** | ***1.18*** |
| ***Heart*** | ***0.90*** | ***0.95*** |  | ***0.88*** | ***1.35*** | ***1.32*** | ***1.34*** | ***1.22*** |  | ***1.23*** | ***1.20*** |
| ***Liver*** | ***0.94*** | ***0.87*** |  | ***0.95*** | ***1.34*** | ***1.33*** | ***1.38*** | ***1.21*** |  | ***1.20*** | ***1.22*** |
| ***Lkidney*** | ***0.92*** | ***0.85*** |  | ***0.95*** | ***1.33*** | ***1.29*** | ***1.32*** | ***1.19*** |  | ***1.16*** | ***1.21*** |
| ***Rkidney*** | ***0.89*** | ***0.85*** |  | ***0.95*** | ***1.36*** | ***1.31*** | ***1.37*** | ***1.20*** |  | ***1.18*** | ***1.22*** |
| ***Tumour*** | ***12.22*** | ***12.05*** |  | ***12.57*** | ***20.67*** | ***20.53*** | ***20.73*** | ***16.75*** |  | ***16.92*** | ***17.22*** |

Reviewer #2: I find this work to be accessible, well defined, and nice to read. Below are some comments.

1. I think that figure 3 would benefit from adding a drawind which more clearly shows how the phantom and the film is located relative to the irradiation geometry.
Thank you for the valuable comments. A cutaway drawing of the phantom irradiation was added to figure 3 as below and the description was given in the caption (D) in lines 211-214.

***“D) Cutaway drawing of the collimator with the PMMA phantom positioned at the midplane of the collimator and the film dosimeter sandwiched between phantom slabs irradiated with Cs-137 beam. Film dosimeter, phantom slabs, and the collimator’s components are marked.”***

2. I think table 1 should also include max values.

We agree that the maximum dose values should be reported. This was requested by the reviewer 1 too. So, the maximum dose values have been already added to table 2.

3. I would like an indication in the graph areas (or headers) of figure 8 that makes it immediately obvious which graph represents which tumour size.

Headers with tumour sizes were added to the graph and figure 8 was updated in the manuscript (below).

Line 221: I would like you to include the equations for the gamma evaluation.

The gamma index equation along with its explanation (below) were added to the manuscript in line 232-237.

***“…quantity through the following equation, known as the Gamma value [56].***

$\boldsymbol{\gamma}\left( {\vec{\boldsymbol{r}}}_{\boldsymbol{ref}}\boldsymbol{,}{\vec{\boldsymbol{r}}}_{\boldsymbol{m}} \right)\boldsymbol{=}\min\left\{ \sqrt{\frac{\boldsymbol{|}{{\vec{\boldsymbol{r}}}_{\boldsymbol{ref}}\boldsymbol{-}{\vec{\boldsymbol{r}}}_{\boldsymbol{m}}\boldsymbol{|}}^{\boldsymbol{2}}}{\boldsymbol{DTA}^{\boldsymbol{2}}}\boldsymbol{+}\frac{{{\boldsymbol{|D (}\vec{\boldsymbol{r}}}_{\boldsymbol{ref}}\boldsymbol{)-D(}{\vec{\boldsymbol{r}}}_{\boldsymbol{m}}\boldsymbol{)|}}^{\boldsymbol{2}}}{\boldsymbol{\Delta}\boldsymbol{D}^{\boldsymbol{2}}}} \right\}$	***(1)***

***where***

${\boldsymbol{|D (}\vec{\boldsymbol{r}}}_{\boldsymbol{ref}}\boldsymbol{)-D(}{\vec{\boldsymbol{r}}}_{\boldsymbol{m}}\boldsymbol{)|}$***is the dose difference and*** ${\boldsymbol{|}\vec{\boldsymbol{r}}}_{\boldsymbol{ref}}\boldsymbol{-}{\vec{\boldsymbol{r}}}_{\boldsymbol{m}}\boldsymbol{|}$ ***is the distance between the dose points. “***

Line 227: I think 3mm and 3% is high, especially with respect to the dimensions of your experiment. I recommend that you redo your evaluation with 2mm and 2%, which I believe is also according to the latest recommendations from AAPM.

We agree with the reviewer. The gamma analysis has been performed also for 2 mm and 2% limits and the corresponding gamma analysis plot (Fig 5) has been updated in the manuscript (shown below).

Line 279: Could you comment on the deviations between the dose curves of fig. 5?

To address the comment the following statement was added to the manuscript in lines 298-300. (Please note that the corresponding detailed discussion is also present in the discussion section).

***“The major deviations between the dose profiles might arise from the statistical nature of the MC simulation as well as uncertainties in the GAFchromic film dosimetry as detailed in the discussion section.”***

Line 285: What are standard acceptance criteria for gamma test and how do your results relate to that?

Gamma values of less than 1 imply that the gamma test was passed. This has been already mentioned in the material and method section. This was restated in the result section lines 297-299 to address the comment as below:

***“******Only two gamma values were smaller than 1 and therefore failed the gamma test.”***

Line 320: I believe you mean 8A, 8B and 8C.

This was corrected in line 335 following the comment.

Line 331-332: This refers to mean values. Update when you add max values.

The values were updated in lines: 42, 347, 396, 426, 431, 461.

Line 391: The challenge of homogeneous dose would be expected before the experiment. How come you did not include bolus as you suggest?

To explain why the bolus was not included for irradiation in this study the following statement was added to the discussion section in lines 420 to 424. Also, two new references (64 and 65) were included to support the statement.

***“It must be highlighted that our specific study ultimately aims at investigating abscopal effects of RT [45], for which we do not need to deliver a homogeneous dose to the whole target [44], but only deliver enough dose to a certain fraction of the target [64], [65]. So, at this stage, it was not essential to employ a bolus for tumour irradiations*** ***in our experiments.”***

Line 413-414: This statement is true only within the range of studied tumour sizes. Either add this remark or weaken the statement of validity.

The remark recommended by the reviewer was added to the manuscript in lines 435-437.

***“Based on this, the dosimetric verifications performed in the experiments are valid at all stages of mice irradiations, when tumours grow within the range of the investigated tumour dimensions.”***

Reviewer #3: The paper entitled " Absorbed Dose Calculation for a Realistic CT-derived Mouse Phantom Irradiated with a Standard Cs-137 Cell Irradiator Using a Monte Carlo Method" has been reviewed for a potential publication in Journal PLOS ONE. The authors develop a CT-derived MC phantom generated from a mouse with a xenograft tumour that used to calculate both the dose heterogeneity in the tumour volume and out of field scattered dose for pre-clinical small animal irradiation experiments. This theoretical method is providing extensive data for the scientific community. Overall the paper is interesting. Most importantly, to ensure that the tumor is treated with the desired dose and that the dose to normal tissue (out of a field) has minimal impact. The titles along with their subtitles are prepared in a proper way.
I believe that the provided outcomes would enhance the existing data for preclinical radiobiological research.
However, I have the following recommendations for the authors, which will improve the overall quality of the paper.

* 16, 17 , 19, 21 , 22. 23, 24, 25, 26, 29, 30, 31, 38, 56, 57,58, 62 and 63 references need to be updated. They are all 2010 and older.

Thank you for the valuable comment which helps us to improve the overall quality of the paper. The intended references were updated to 2010 and more recent publications in the manuscript as follows:

***16. Xie T, Zaidi H. Development of computational small animal models and their applications in preclinical imaging and therapy research. Medical Physics. 2016 Jan;43(1):111-31.***

***17. Kainz W, Neufeld E, Bolch WE, Graff CG, Kim CH, Kuster N, Lloyd B, Morrison T, Segars P, Yeom YS, Zankl M. Advances in computational human phantoms and their applications in biomedical engineering—a topical review. IEEE transactions on radiation and plasma medical sciences. 2018 Dec 3;3(1):1-23.***

***19. Xu XG. An exponential growth of computational phantom research in radiation protection, imaging, and radiotherapy: a review of the fifty-year history. Physics in Medicine & Biology. 2014 Aug 21;59(18):R233.***

***21. Xie T, Zaidi H. Development of computational small animal models and their applications in preclinical imaging and therapy research. Medical Physics. 2016 Jan;43(1):111-31.***

***22. Xie T, Zaidi H, Segars P. Design and construction of computational animal models. InComputational Anatomical Animal Models: Methodological developments and research applications 2018 Dec 1. IOP Publishing.***

***23. Lee CL, Park SJ, Jeon PH, Jo BD, Kim HJ. Dosimetry in small-animal CT using Monte Carlo simulations. Journal of Instrumentation. 2016 Jan 27;11(01):T01003.***

***24. Papadimitroulas P, Kagadis GC. Applications of computational animal models in ionizing radiation dosimetry. InComputational Anatomical Animal Models: Methodological developments and research applications 2018 Dec 1. IOP Publishing.***

***25. Perrot Y, Maigne L, Breton V, Donnarieix D. Validation of GATE 6.1 for targeted radiotherapy of metastic melanoma using 131I-labeled benzamide. InThird European Workshop on Monte Carlo Treatment Planning 2012 May 15 (pp. 5-p).***

***26. Vasquez SX, Shah N, Hoberman AM. Small animal imaging and examination by micro-CT. InTeratogenicity testing 2013 (pp. 223-231). Humana Press, Totowa, NJ.***

***29. Praveenkumar RD, Santhosh KP, Augustine A. Estimation of inhomogenity correction factors for a Co-60 beam using Monte Carlo simulation. Journal of Cancer Research and Therapeutics. 2011 Jul 1;7(3):308.***

***30. De Marzi L, Lesven C, Ferrand R, Sage J, Boulé T, Mazal A. Calibration of CT Hounsfield units for proton therapy treatment planning: use of kilovoltage and megavoltage images and comparison of parameterized methods. Physics in Medicine & Biology. 2013 May 29;58(12):4255.***

***31. Schreuder AN, Bridges DS, Rigsby L, Blakey M, Janson M, Hedrick SG, Wilkinson JB. Validation of the RayStation Monte Carlo dose calculation algorithm using realistic animal tissue phantoms. Journal of Applied Clinical Medical Physics. 2019 Oct;20(10):160-71.***

***38. Wiklund K, Toma-Dasu I, Lind BK. The influence of dose heterogeneity on tumour control probability in fractionated radiation therapy. Physics in Medicine & Biology. 2011 Nov 15;56(23):7585.***

***59. Niko H, Dafina X, Theodhor K, Ervis T. Calculation Methods in Radiotherapy Using MATLAB. Journal International Environmental Application Science, ISSN. 2014 Jun 1:1307-0428.***

***60. Baiker M, Milles J, Dijkstra J, Henning TD, Weber AW, Que I, Kaijzel EL, Löwik CW, Reiber JH, Lelieveldt BP. Atlas-based whole-body segmentation of mice from low-contrast Micro-CT data. Medical image analysis. 2010 Dec 1;14(6):723-37.***

***67. Formenti SC. Optimizing dose per fraction: a new chapter in the story of the abscopal effect?. International Journal of Radiation Oncology, Biology, Physics. 2017 Nov 1;99(3):677-9.***

***68. Drissi R, Wu J, Hu Y, Bockhold C, Dome JS. Telomere shortening alters the kinetics of the DNA damage response after ionizing radiation in human cells. Cancer Prevention Research. 2011 Dec;4(12):1973-81.***

Reviewer #4: The paper is well written and structured and the conclusions are well supported by the experimental results and calculations. However, I think that the authors should be more stringent about the radiation dose quantity used. The term “dose” is used in the manuscript and this ought to be replaced by “absorbed dose”.

Thank you for the comments. To address this comment, the term “dose” was replaced by “absorbed dose” in lines: 39, 83, 130, 316, 346, 363, 365, 402, 409 throughout the manuscript.

Specific comments:
Line 247: The second “right lung” should be “right kidney”.

“right lung” was replaced by “right kidney” in Line 260.

Line 320: I guess that it should be Figure 8A, 8B, 8C.

This was corrected in line 335 following the comment.
