# Supplementary material for: Absorbed dose calculation for a realistic CT-derived mouse phantom irradiated with a standard Cs-137 cell irradiator using a Monte Carlo method
Source: PLoS One. 2023 Feb 2;18(2):e0280765. doi: 10.1371/journal.pone.0280765 (PMC9928120; doi:10.1371/journal.pone.0280765)
Supplement: Attachment — Submitted filename: Response to Reviewer .docx [file pone.0280765.s004.docx]

We appreciate the editor and the reviewer for taking the time to read and comment on our manuscript. The comments from the reviewer-4 are in black and our responses are highlighted in red. Also, the changes that we have made to the manuscript are all ***in bold italic text*** in both response letter and the revised manuscript. With the exceptions of exchanging the reference numbers 56 and 57 and correcting the reference number 6 in the text, we did not make any changes to the reference list.

Please note that this is the second letter of “Response to Reviewer” for our submission. In the first round of the revision (with decision of “MINOR REVISION”), we addressed all comments from the reviewers 1, 2, 3, and 5. So, in the present revision only the comments of reviewer 4 have been addressed.

**Comments**

1. Lines 180-206; Due to the nature of complexity of the mouse phantom, which uses different materials representing the air, lung, soft tissue and bone materials within the phantom, the choice of a single, simple PMMA phantom for verification could be interpreted as a bit weak in my opinion. Especially, as the PMMA material is not utilised in the actual mouse phantom. I would suggest either finding an additional physical phantom of at least of one of the materials used within the mouse phantom for additional verification, or using other means to strengthen the sufficiency of verification using only PMMA phantom – for example references.

Thank you for the valuable comments which helps us to improve the paper.

Verification of the Monte Carlo modelling is commonly fulfilled by comparing the measured and calculated dose distributions. Many studies use dosimeters within “slabs of PMMA phantoms” or “simple water phantoms” to measure the doses and compare them with the MC calculated results. This indicates that the utilization of “simple PMMA phantoms” for dose measurements and comparing the measured doses with the MC calculated results is a valid verification method as we used in this study. To address the comment, the following statement was added to the discussion section (lines 379-382) and the relevant references were provided (as suggested by the reviewer).

***“It is worth mentioning that several studies have used the same method for verifying MC simulations [55], [32], [51], which confirms the validity of the verification method used in this study, i.e., comparing the doses measured within a PMMA phantom with the corresponding calculated MC doses.”***

2. Why not using a simpler way to analyse the data? Either way, the data obtained from
   the Gafchromic film is 2D, so just comparing 2D dose maps with some particular selected values would be enough, as in this 2D situation, the choice of the Gamma index analysis does not seem to provide significant advantages over a simple 2D dose map comparison. Although, if there are such advantages, this should be mentioned in the running text.

Since Gamma analysis is the gold standard for dose distribution comparisons, authors decided to perform the gamma index analysis for a robust MC simulation verification in this study. So, to address the comment, the advantage of the gamma index analysis along with the relevant reference to support the statement were added to the manuscript in lines 228-231 as follows:

***“Gamma analysis is the most effective method for comparing two dose distributions [56]. It is established as the gold standard in verification procedures among all available methods and clinical decisions are made based on its outcomes [56], [57].*** “

1. In a case, where the Gamma index analysis is sufficiently substantiated by the authors, I believe there should be somewhat more explanation within the running text about the Gamma index. At least some adapted figures explaining the basic principles of the method, which then the reference to the work by Diamantopoulos et al. would complete.

The complete equation of the gamma and its corresponding explanations (see equation 1 below) were added to the manuscript based on comment from the reviewer 2 in the first round of revision submission as follows:

unitless quantity through the following equation, known as the Gamma value [56].

$\gamma\left( \vec{r}_{\mathrm{ref}}, \vec{r}_{m} \right)=\min\left\{ \sqrt{\frac{|{\vec{r}_{\mathrm{ref}}-\vec{r}_{m}|}^{2}}{\mathrm{DTA}^{2}}+ \frac{{{|D (\vec{r}}_{\mathrm{ref}})-D(\vec{r}_{m})|}^{2}}{\Delta D^{2}}} \right\}$	(1)

where

${|D (\vec{r}}_{\mathrm{ref}})-D(\vec{r}_{m})|$is the dose difference and ${|\vec{r}}_{\mathrm{ref}}-\vec{r}_{m}|$ is the distance between the dose points.

To address the comment, the following explanations were added to the manuscript (lines 240-243, next to the Gamma equation) to provide more details on the basic principles of the Gamma analysis as requested.

***“In this way, an acceptance region (an ellipse) is created around every single point of the reference dose distribution. This ellipse should compass the evaluated dose point to pass the gamma test. Mathematically, gamma values of less than 1 imply that the gamma test was passed [56].”***

4. Line 295; All doses were normalised to the depth of maximum dose? Following text suggest that the Dmax means the maximum dose. Please clarify.

Thank you for the significant comment. D_max_ is maximum dose. The text was corrected following the comment in line 315.

5. Lines 317-329; Differences within the different DVH curves are discussed in the running text. It would be much clearer if the curves would be plotted on the same graph, so that the differences would be more apparent to the reader.

We agree with the reviewer that plotting all DVHs on the same graph contributes to the clarity. So, to address the comment, the DVHs were plotted on a single graph and figure 8 was updated in the manuscript (see below). Also, the figure number were updated in the manuscript (in lines 340, 356, 357) and the caption of the figure 8 was modified in lines 359-361.

***“Fig 8. DVHs plots. DVHs of the tumours and OARs for 0.5 cm, 0.75 cm, and 1 cm tumour irradiations are demonstrated in dash, dot, and solid lines, respectively. DVHs of the tumours are in black and colours of the DVHs for OARs are presented in the graph.”***

6. Fig 2, mistakes within the text in figure, like “Virtual Cs-137 sorce” and “used at beam angel”.

Thank you for your scrutiny. Mistakes in the figure were corrected and the figure 2 was updated in the manuscript as below.
