# Supplementary material for: Absorbed dose calculation for a realistic CT-derived mouse phantom irradiated with a standard Cs-137 cell irradiator using a Monte Carlo method
Source: PLoS One. 2023 Feb 2;18(2):e0280765. doi: 10.1371/journal.pone.0280765 (PMC9928120; doi:10.1371/journal.pone.0280765)
Supplement: Attachment — Submitted filename: Response to Reviewers.docx [file pone.0280765.s005.docx]

We appreciate the Editor for taking the time to read and comment on our manuscript. The comments from the Editor are in black and our responses are highlighted in red. Also, the changes that we have made to the manuscript are all ***in bold italic text*** in both response letter and the revised manuscript.

Reference 32, 50, 56, 65, 66, 76, 68, 69, 70, 71, 72, and 73 were added to the manuscript. Accordingly, reference numbers 39-82 were updated throughout the manuscript.

Please note that this is the third letter of “Response to Reviewers” for our submission. In the last rounds of the revisions (with decision of “MINOR REVISION”), we addressed all comments from the reviewers 1, 2, 3, 4, and 5. So, in the present revision only the comments of the Editor have been addressed.

Additional Editor Comments:
Thanks to the authors for the revised mansucript. Before we proceed to accept the submited work for publications, there are few minor corrections are needed to improve the quality of the submitted work. Kinldy find my following comments for your references.

1. Line 33 please name the phantom.

Thank you for the valuable comments which help us improve the paper. The name of the phantom has been added in line 33.

2. Line 94-97: This is the specific objectives of the work and in Line 101-103 again, the author mentioned the general motivation of this work. Please revise those lines to make avoid redundant and confused the reader.

The redundant information in lines 101-103 has been removed.

3. Line 98-101: This should be in methodology to address Pb colimator consideration for efficient irradiations. Please remove from Introduction.

The details of the collimator have been removed from the Introduction as suggested and have been added to the methodology section with minor changes as below (lines 136-142).

***“We have previously constructed an add-on lead collimator to allow for targeted irradiation of mice [51]. In previous studies, we showed that our collimator combined with a 137Cs source irradiator is an effective method for irradiating small animal xenograft tumours [51], [52], [53]. The simulation of MC mouse phantom irradiation was performed using a 1-cm diameter beam produced by the collimator [51] mounted onto a Gammacell 40 Exactor irradiator unit (Best Theratronics, ON K2K OE4, Canada) located at Transitional Research Institute (TRI), Brisbane, Australia.”***

4. Line 105-109: The importance or the finding from this study should be in early part of introduction not in the last para of introduction.

Thank you for the important comment. The importance of the study has been moved to an earlier part of the Introduction section (lines 89-94).

5. Line 168-170: Please elaborate the importance of these cut-offs configutrations to MC computations.

Thank you for the comment. Based on the literature, the selected cut-offs allow for an efficient dose calculation while reducing the CPU calculation time. To address the comment the following explanation along with the relevant references were added to the manuscript in lines 180-183.

***“These well-established cut-off settings allow for efficient dose calculations while reducing the CPU calculation time [50]. “***

6. Under 2.2.2 & 2.3.1: Please include the description of secondary electrons transports and other photon-electron interaction treatments for MC computation.

Thank you for the important comment. The missing information about the secondary electrons transport and photon-electron interactions are provided in lines 173-181 and 236-237 under 2.2.2 and 2.3.2, respectively, as suggested by the Editor.

lines 173-180

***“******Electron impact ionization, atomic relaxations, and low energy photon interactions, such as Rayleigh scattering and bound Compton scattering, have been included in our MC simulations [55], [56]. Also, through the condensed history technique, catastrophic inelastic scattering and bremsstrahlung were taken into account in the transport of secondary electrons [56]. Global Electron transport cut-offs (ECUT) and Global photon transport cut-offs (PCUT) were 0.521 MeV and 0.01 MeV respectively [54]. Also, the low energy thresholds for electrons and photons AE and AP were set to 0.521 and 0.01, consistent with the ECUT and PCUT parameters, respectively.”***

lines 236-237

***“The interactions explained in section 2.2.2 were also included in DOSXYZnrc simulations [58]."***

7. Line 104-105: This is the important aspect that need to be focus in introduction. Throughout the Introduction Line 48-85, the authors are addressing the history of mc and ct-based ocmputational phantom, importance of absosrbed dose investigations,problems statetement ie. no available dosimetry study on xenographic tumour dimensions, literature works. Based on the given title, the author should highlight the literature review on how the experimental verification of dosimetry onthe proposed phantom was conducted in the past?

As suggested by the Editor, to highlight the literature review on how the verification of MC modelling/simulations has been conducted in the past we performed a systematic literature review, using the PRISMA diagram (shown below).

Systematic PRISMA literature review details

1.	Database: PubMed

Keyword: “PMMA phantom” AND "dose measurement" AND Monte Carlo AND (radiotherapy OR Irradiation) AND “small animal”. This includes all the search terms in the different combinations using Boolean operators (AND or OR).

Main limits: (years of search >2000, English language).

2.	Articles that appear more than once were manually removed.

3.	Titles and abstracts for articles that are relevant to this research were screened. All 28 articles appeared to provide information related to the research. As a result, 0 articles excluded based on this screening.

4.	After reading them, 13 articles not exactly relevant to the context were excluded.

5.	As the final step, we subtracted the number of excluded articles or records during the eligibility review of full texts from the total number of articles reviewed for eligibility. Finally, the PRISMA flow diagram completed with "15" articles for literature review. Only 6 studies presented exactly the same method of PMMA irradiations for the MC simulation verifications. Explanations about these 6 studies are included in the manuscript.

15 articles on the small animal phantom dosimetry using MC simulations have been systematically reviewed. The literature review gives rise to the fact that verification of the Monte Carlo modelling is commonly fulfilled by comparing the measured and calculated dose distributions using “slabs of PMMA phantoms” or “simple water phantoms. Similar to our study, 6 studies have used dosimeters within “slabs of PMMA phantoms” to measure the doses and compare them with the MC calculated results. To address the comment, the brief literature review was added to the introduction (lines 104-110) and the relevant references were provided (as suggested by the Editor) as below.

***“Verification of Monte Carlo simulations is commonly accomplished by comparing dose distributions obtained from physical dose measurements with the corresponding calculated doses.*** ***Good agreement between the measured and calculated doses guarantees the accuracy of MC modelling. Several studies have used dosimeters within slabs of PMMA phantom or simple water phantoms to measure the doses, comparing them with the MC simulation results [47], [48], [49], [50], [51], [52]. In this study, we use the same method for the verification of our MC simulations.”***

8. Line 180: Please extend th e discussion why PMMA is chosen over other tissue weighted equivalent materials i.e nylon, polyacetate? Indicate the composition, density and compare to other tissue eg bone, adipose, muscle etc. as well as reference study that using PMMA for dosimetry verifcation of MC phantom study. Any approximation need to be stated in this part fopr the considertation of PMMA.

Thank you for the comment. Firstly, as it was shown in our systematic literature review, several studies used PMMA phantom irradiations to verify MC modelling. This indicates that the utilization of “simple PMMA phantoms” for dose measurements and comparing the measured doses with the MC calculated results is a valid verification method as we used in this study.

Secondly, PMMA is chosen over other materials because it is tissue equivalent, has a reproducible composition, and easy to machine. Also, PMMA phantoms are employed for standard dosimetry practices like calibration of in-phantom dose rate (relevant references were given). The PMMA phantom well mimics the soft tissue composition and therefore it effectively approximates the tissue of the xenograft tumour.

Thirdly, one reference was given in order to provide requested information on the PMMA phantom composition and electron density.

Lastly, one reference was given in which the mouse tissues were considered PMMA equivalent for dosimetric verification purposes.

To address the comment the following statement is added to the discussion section in lines 401-413 as below:

***“As mentioned in the introduction section, several studies have used the same method for verifying MC simulations. This provides strong support for the validity of the verification method used in this study, i.e., comparing the doses measured within a PMMA phantom with the corresponding calculated MC doses. It is worth mentioning that we used PMMA material in this study because it is tissue equivalent, has a reproducible composition, is easy to machine, and it is commonly used for radiation dosimetry investigations [65], [66], [67], [68], [69]. Also, PMMA phantoms are typically employed for standard dosimetry practices such as calibration of in-phantom dose rate [70], [71]. PMMA is found to be an excellent soft-tissue substitute as its material properties such as effective atomic number and electronic density are close to that of soft tissue [72]. So, the in-house built PMMA phantom well mimics soft tissue composition of the mouse. Similar to our method, in research conducted by Kuess et al [73] the mouse tissues were considered PMMA equivalent for dosimetric verification purposes.”***

9. Under 2.3.1, Line 190 : Please extend the discussion on the approach and technique for quality control of Gafchromic EBT3 film measurement? any other solid state dosimeters used for dose corrections?

Thank you for the comment. To ensure the accuracy of the Gafchromic EBT3 film measurement, film dosimetry was performed based on the Gafchromic film dosimetry procedure given in previous studies (as has been already mentioned in section 2.3.1 lines 213-214. Subsequently, the measured doses were compared against the corresponding doses obtained from MOSKIN dosimeter for dose corrections. To address the comment the following statement was added to the discussion section line 425-428.

***“To ensure the accuracy of our Gafchromic EBT3 film measurement, the measured doses were compared against the corresponding doses obtained from MOSKIN dosimeter as detailed in our previous study [51].”***

10. Result, 3.1: Please include a table of data for Figure 5A. The given tabulated data will benefit the reader apart of it is easier for evaluator to check the disparity of dose over x for both MC data vs Exp. PMMA doses. Im wondering the variation of the error of MC computations for different parameters, not sure I have mislook on the error figure in discussion. Please include the value in this table as well as the formula for relative errors estimations.

A table containing the measured and calculated data was included to the manuscript to demonstrate the disparity of doses for each point of MC calculation and the corresponding measured points (see below). Also, the errors for all calculated points were obtained from the 3ddose file and added to the table. The following statement along with the table was added to the manuscript (lines 311-314).

***“******The MC calculated doses for all points along with the corresponding measured doses, corresponding to the profiles shown in figure 5A, are presented in Table 1. Also, the errors for all MC calculated doses were obtained from the 3ddose file and presented in the last column of the table.”***

***Table 1. Measured and calculated doses and MC dose calculation errors.***

| ***Distance (cm)*** | ***Measured doses (%)*** | ***MC Calculated doses (%)*** | ***MC calculation errors (%)*** |
| --- | --- | --- | --- |
| ***0*** | ***100*** | ***100*** | ***0.88*** |
| ***0.12*** | ***99.44*** | ***97.53*** | ***0.79*** |
| ***0.22*** | ***97.62*** | ***98.13*** | ***0.65*** |
| ***0.32*** | ***94.05*** | ***99.75*** | ***1.18*** |
| ***0.42*** | ***82.37*** | ***90.42*** | ***0.78*** |
| ***0.52*** | ***62.30*** | ***62.64*** | ***0.85*** |
| ***0.62*** | ***34.74*** | ***35.31*** | ***1.62*** |
| ***0.72*** | ***15.73*** | ***14.32*** | ***1.92*** |
| ***0.82*** | ***13.02*** | ***11.85*** | ***2.10*** |
| ***0.92*** | ***10.57*** | ***10.61*** | ***2.92*** |
| ***1.02*** | ***10.45*** | ***11.12*** | ***2.79*** |
| ***1.12*** | ***11.20*** | ***11.48*** | ***3.29*** |
| ***1.22*** | ***9.61*** | ***9.82*** | ***2.91*** |
| ***1.32*** | ***9.60*** | ***9.01*** | ***3.22*** |
| ***1.42*** | ***9.66*** | ***8.61*** | ***2.99*** |
| ***1.52*** | ***9.09*** | ***8.73*** | ***2.82*** |
| ***1.62*** | ***9.60*** | ***8.67*** | ***3.30*** |
| ***1.72*** | ***9.50*** | ***8.94*** | ***1.98*** |
| ***1.82*** | ***9.38*** | ***9.02*** | ***3.21*** |
| ***1.92*** | ***9.43*** | ***8.92*** | ***2.96*** |
| ***2.02*** | ***9.46*** | ***7.39*** | ***3.34*** |
| ***2.12*** | ***9.54*** | ***7.40*** | ***2.92*** |
| ***2.22*** | ***9.41*** | ***7.13*** | ***3.07*** |
| ***2.32*** | ***9.32*** | ***6.82*** | ***2.82*** |
| ***2.50*** | ***9.39*** | ***6.60*** | ***2.98*** |

***Dose values for all points of the calculated profile along with the corresponding measured doses (normalized to their respective maximum doses). Dose calculation errors are presented in the last column.***
